# Time-Resolved Molecular Characterization of Secondary Organic Aerosol Formed from OH and NO_3_ Radical Initiated Oxidation of a Mixture of Aromatic Precursors

**DOI:** 10.1021/acs.est.3c00225

**Published:** 2023-07-27

**Authors:** Varun Kumar, Jay G. Slowik, Urs Baltensperger, Andre S. H. Prevot, David M. Bell

**Affiliations:** Laboratory of Atmospheric Chemistry, Paul Scherrer Institute (PSI), Villigen 5232, Switzerland

**Keywords:** secondary organic aerosol, aromatic VOCs, intra-particle reactions, aromatic oxidation, SOA composition, extractive electrospray ionization

## Abstract

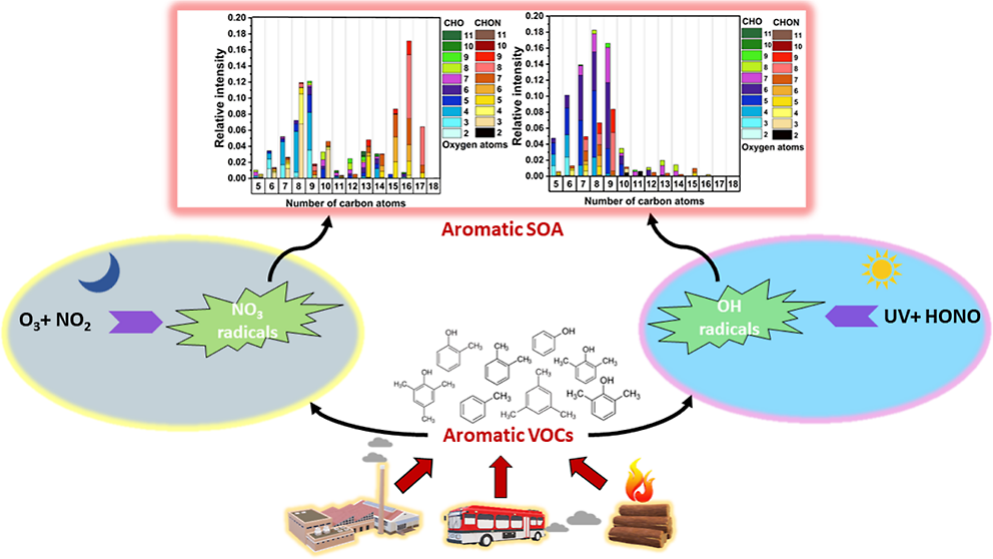

Aromatic hydrocarbons (ArHCs) and oxygenated aromatic hydrocarbons (ArHC–OHs) are emitted from a variety of anthropogenic activities and are important precursors of secondary organic aerosol (SOA) in urban areas. Here, we analyzed and compared the composition of SOA formed from the oxidation of a mixture of aromatic VOCs by OH and NO_3_ radicals. The VOC mixture was composed of toluene (C_7_H_8_), *p*-xylene + ethylbenzene (C_8_H_10_), 1,3,5-trimethylbenzene (C_9_H_12_), phenol (C_6_H_6_O), cresol (C_7_H_8_O), 2,6-dimethylphenol (C_8_H_10_O), and 2,4,6-trimethylphenol (C_9_H_12_O) in a proportion where the aromatic VOCs were chosen to approximate day-time traffic-related emissions in Delhi, and the aromatic alcohols make up 20% of the mixture. These VOCs are prominent in other cities as well, including those influenced by biomass combustion. In the NO_3_ experiments, large contributions from C_*x*_H_*y*_O_*z*_N dimers (C_15_–C_18_) were observed, corresponding to fast SOA formation within 15–20 min after the start of chemistry. Additionally, the dimers were a mixture of different combinations of the initial VOCs, highlighting the importance of exploring SOAs from mixed VOC systems. In contrast, the experiments with OH radicals yielded gradual SOA mass formation, with C_*x*_H_*y*_O_*z*_ monomers (C_6_–C_9_) being the dominant constituents. The evolution of SOA composition with time was tracked and a fast degradation of dimers was observed in the NO_3_ experiments, with concurrent formation of monomer species. The rates of dimer decomposition in NO_3_ SOA were ∼2–3 times higher compared to those previously determined for α-pinene + O_3_ SOA, highlighting the dependence of particle-phase reactions on VOC precursors and oxidants. In contrast, the SOA produced in the OH experiments did not dramatically change over the same time frame. No measurable effects of humidity were observed on the composition and evolution of SOA.

## Abstract 2

This study examines the composition of SOA formed by the reactions of a mixture of aromatic VOCs with OH and NO_3_ radicals, as well as the role of intra-particle reactions in the evolution of SOA composition.

## 1 Introduction

In urban areas, anthropogenic volatile organic compounds (VOCs), emitted by vehicular exhaust, biomass burning, and use of solid fuels,^2,3^ are a major fraction of the total VOCs present.^1,4,5^ The main contributors to anthropogenic VOCs present in these environments are aromatic hydrocarbons such as benzene (C_6_H_6_), toluene (C_7_H_8_), ethylbenzene (C_8_H_10_), and tri-methylbenzene (C_9_H_12_), which are henceforth termed as ArHCs in this study. ArHCs and their associated alcohols (denoted ArHC–OHs here) can contribute to urban O_3_ pollution,^6,7^ photochemical smog,^8^ and secondary organic aerosol (SOA) formation.^9,10^ In addition, the SOA formed from oxidation of these precursors is known to have adverse effects on human health.^11−13^ It has been shown that the oxidative potential (OP) of particulate matter (PM) (i.e., the ability of particles to generate reactive oxygen species (ROS) and create an imbalance in the favor of oxidants) depends on both its concentration and composition,^14−16^ with SOA identified as an important contributor to OP. Recently, the non-exhaust vehicular emissions and anthropogenic SOA were shown to be the largest contributors to aerosol OP throughout most of Europe.^17^ These findings support the observations in Delhi,^18^ where the OP was highest during the afternoon period when the PM mass is dominated by SOA formed from aromatic precursors.^19^ These observations suggest a link between the SOA formed from the anthropogenic precursors and increased health risks.

The oxidation of anthropogenic VOCs in the atmosphere is driven by the initial attack of an oxidant (OH or NO_3_) on the parent VOC followed by addition of O_2_, which generally results in the formation of a peroxy (RO_2_) radical.^20−22^ The fate of these RO_2_ radicals depends upon the concentration of NO_*x*_ (NO + NO_2_) and other radicals present in the atmosphere (e.g., HO_2_, RO_2_ and NO_3_). In a low-NO_*x*_ regime, RO_2_ radicals predominantly react with HO_2_ and other RO_2_ radicals (including self-reactions), or undergo autoxidation to form highly oxygenated molecules.^23−25^ In a high-NO_*x*_ regime, RO_2_ radicals predominantly react with NO to produce organo-nitrates (RONO_2_) or proceed to form alkoxy radicals (RO) which fragment into smaller molecules, thereby reducing the amount of SOA formed.^26,27^ Depending upon the fate of RO_2_ radicals, different oxidation products may be produced in the gas phase, some of which may then partition into the particle phase to form SOA.

Recent studies of α-pinene SOA have shown that the composition of SOA is not solely governed by gas-particle partitioning, but additionally by particle-phase reactions, which include reactions under dark conditions.^28,29^ These reactions alter the chemical composition of the particles and may alter the physical properties of SOA. The changes brought about by the particle-phase reactions can be rapid, for example, half-lives of some decaying species are less than an hour.^28^ In addition, the ROS such as peroxides, which are linked to oxidative stress and detrimental health effects, have been shown to be reactive and have short lifetimes.^30,31^ Owing to these reasons, it becomes essential to conduct highly time-resolved measurements to study the changes occurring on timescales of minutes to hours in chamber/laboratory settings and accurately associate them with comparable atmospherically relevant time scales to better constrain the health effects and chemical properties of SOA.

Chamber studies focused on the oxidation of ArHCs have mostly concentrated on mechanistic and compositional aspects of SOA from single-component aromatic systems.^9,32−34^ The atmosphere, however, comprises a mixture of different VOCs that oxidize simultaneously. The oxidation of a mixture of VOC precursors, together, may produce oxidation products from cross-reactions of RO_2_ radicals produced from different initial VOCs, thus leading to a different SOA composition than that expected from oxidation of single VOC precursors or a simple summation of their respective products.^35,36^ Product interactions may produce SOA with different physical and chemical properties as compared to a single-component SOA.^35,37−39^ Therefore, studying the oxidation of mixtures of VOC precursors will better represent the composition of SOA in urban areas. Additionally, as OH and NO_3_ radicals dominate the daytime and nighttime oxidation, respectively, of these VOCs, a comparison between SOA composition produced by OH and NO_3_ chemistry can provide valuable insights.

Here, we used an extractive electrospray ionization mass spectrometer (EESI-TOF) and an aerosol mass spectrometer (AMS) to measure the bulk composition of SOA formed from the oxidation of an aromatic mixture (including ArHC and ArHC–OH molecules) by either OH or NO_3_ radicals. The high time resolution of the EESI-TOF makes it possible to follow the changes in SOA composition in real-time, determining the processes driving compositional changes. We also compared the rates of decay of some high-molecular weight species observed in the aromatic + NO_3_ system in this study with those observed in the α-pinene + O_3_ system.

## 2 Methodology

### 2.1 Instrumentation

The Aerodyne AMS^40,41^ was used to obtain quantitative measurements of the size-resolved composition of non-refractory (NR; species that flash-vaporize at 600 °C) PM at high time resolution. Here, the sampled aerosol particles pass through an aerodynamic lens and get focused into a particle beam which impact on a heated tungsten surface (∼600 °C, and ∼10^–7^ Torr) and the NR components flash-vaporize. The resulting vapors are ionized by electron ionization (EI, ∼70 eV) and analyzed by a TOF mass spectrometer.

The extractive electrospray ionization time-of-flight mass spectrometer (EESI-TOF) measured the near-molecular level composition (i.e., chemical formulae of the molecular ions) of the SOA formed in the chamber.^28,29,42,43^ Here, the aerosol sample is drawn at 1 L min^–1^ through a multi-channel charcoal denuder (69 channels, volume of 38 cm^3^) to remove the gaseous components. After this, the aerosol sample intersects a charged spray of droplets (50:50 water/acetonitrile, doped with 100 ppm of NaI) emitted by an electrospray capillary (2900 V). The soluble components of the sampled aerosol are extracted in the charged droplets. During evaporation and subsequent Coulomb explosion, the analyte molecules are ejected as Na^+^ adducts. These adducts are guided into a commercial time-of-flight mass spectrometer and analyzed according to their *m*/*z*. The average resolution of the mass spectrometer was between 9000 and 10,000 *M*/Δ*M* at *m*/*z* 185 during all experiments. Additional details on AMS and EESI-TOF data analysis are given in Supporting Information S2 and a comparison of EESI-TOF mass flux, AMS and SMPS is given in Figure S2.

### 2.2 Chamber Experiments

A series of experiments was designed to study the composition and evolution of SOA formed from the oxidation of a mixture of aromatic compounds with OH and NO_3_ radicals. Experiments were conducted in the Paul Scherrer Institute (PSI) atmospheric simulation chamber,^44^ which consists of a 9 m^3^ volume (2.5 m × 1.8 m × 1.9 m, L × W × H), 125 μm thick collapsible bag made of fluorinated ethylene propylene. In containers next to the chamber, the following instruments were installed: an O_3_ monitor (Thermo 49c), a NO_*x*_ monitor (Thermo 42c), a proton-transfer reaction time-of-flight mass spectrometer (PTR-MS, Ionicon PTR 8000), a scanning mobility particle sizer (SMPS, TSI model 3938), a high-resolution time-of-flight AMS (Aerodyne Research, Inc.), and an extractive electrospray ionization time-of-flight mass spectrometer (EESI-TOF, Tofwerk AG). Prior to each experiment, the chamber was cleaned overnight by continuously flushing with 40 L min^–1^ of pure air from a zero-air generation system (737-250 series, AADCO Instruments, Inc., USA). Prior to the start of each experiment, the particle number concentration was checked by the SMPS, and if the total number concentration was below 10 cm^–3^, the chamber was deemed clean.

These experiments focused on the oxidation of a mixture of ArHC and ArHC-OH molecules with both NO_3_ radicals and OH radicals at 40–50 and 70–90% relative humidity (RH). A single ArHC + ArHC–OH mixture was used for all experiments, and consisted of toluene (C_7_H_8_), *p*-xylene + ethylbenzene (1:1 (v/v); C_8_H_10_), 1,2,3-tri-methylbenzene and 1,3,5-tri-methylbenzene (1:1 (v/v); C_9_H_12_), phenol (C_6_H_6_O), o-, m- and p-cresol (1:1:1 (v/v); C_7_H_8_O), 2,3-dimethylphenol (C_8_H_10_O), and 2,4,6-trimethylphenol (C_9_H_12_O). This VOC mixture was designed such that the ratios of toluene, *p*-xylene + ethylbenzene, and tri-methylbenzene in this mixture were ∼6:4:1 on a volume-to-volume (v/v) basis and approximately similar to the ratios of these VOCs in the mass spectrum of a traffic-related factor from a recent VOC source apportionment study in Delhi, which dominated the daytime emissions.^1^ Substituted aromatics, that is, phenol, cresol, 2,6-dimethylphenol, and 2,4,6-trimethylphenol, were added in equal amounts and constituted in total ∼20% by volume of this mixture. The approximate concentrations of the VOCs after injection into the chamber are given in Table S1.

The experimental protocols differed between the OH and NO_3_ experiments and are discussed in detail in the Supporting Information S1. Table S2 summarizes the experimental conditions of all experiments conducted. In all experiments, the mass measured by the SMPS was corrected for particle wall loss (Supporting Information S3). A size-dependent wall loss was applied to different species measured by the AMS (Figure S10).

During the course of the OH experiments, there was an integrated OH exposure of 5.67 × 10^7^ molec cm^–3^ h, which is equivalent to about 56.7 h or 2.4 day equivalents of atmospheric aging assuming a global average OH concentration of 1 × 10^6^ molec cm^–3^. Note that this value is taken from Experiment 5 (due to the PTR-MS not operating for the other OH experiments; see Table S2) and assumed to approximately hold for the other OH experiments, given the same lights and identical HONO production. More details on the calculation of OH exposure are given in Supporting Information S4.

The NO_3_ concentrations were modeled with F0AM (see next section) to provide an upper limit on the NO_3_ exposure in the chamber, which under our experimental conditions corresponds to ∼30 days of aging in ∼3 h of experimental time.

### 2.3 Chamber Box Modeling

The Framework for 0-D Atmospheric Modeling (F0AM) with the Master Chemical Mechanism (MCM) was used to model the radical chemistry and reactivity of precursor VOCs in the chamber.^45^ The model incorporates the reactions in the MCM^46,47^ to simulate atmospherically relevant chemical systems. The initial concentrations of VOCs in the chamber were used as model inputs. The model was run with the same initial concentrations of VOCs for simulating both OH and NO_3_ reactions.

## 3 Results and Discussion

In this section, we will only discuss the experiments conducted at 50% RH. The experiments conducted at 90% RH were evaluated to determine whether there were any systematic changes in SOA composition with an increase in RH. As this was not the case (see Figure S1), the high RH experiments are treated here as replicates.

### 3.1 VOC Consumption and SOA Formation

In the experiments conducted here, SOA formation from the same mixture of ArHC and ArHC–OH was probed using either NO_3_ or OH radicals. The reactivity of the VOC mixture depends strongly on the oxidant identity: the ArHC and ArHC–OH molecules used here have reaction rates with OH radicals spanning roughly an order of magnitude (on the order of 10^–11^ to 10^–12^ cm^3^ molec^–1^ s^–1^) while with NO_3_ they differ by almost 5 orders of magnitude (for ArHC ∼ 10^–17^ cm^3^ molec^–1^ s^–1^ and for ArHC–OH ∼ 10^–12^ cm^3^ molec^–1^ s^–1^).^48,49^Figure S3 models the expected reactivity of NO_3_ and OH radicals toward both ArHC and ArHC–OH molecules using F0AM coupled to the MCM (see Section 2.3), which agrees with the observed ArHC and ArHC–OH time series from the PTR-MS (Figure S3). Therefore, the difference in reactivity will result in nearly exclusive reactions of ArHC–OH + NO_3_. In contrast, for the OH experiments, a constant mixing ratio of ∼50 ppbv of HONO in the chamber leads to the model prediction that both ArHC and ArHC–OH will steadily react away, which agrees with the measured VOC consumption and the corresponding calculated OH exposure based on the differential reactivity of *d*_9_-butanol (fragment at mass-to-charge ratio *m*/*z* 66.126, [C_4_D_9_]^+^) and toluene (fragment at *m*/*z* 93.15, [C_7_H_8_]H^+^).^50^

**Figure 1:**
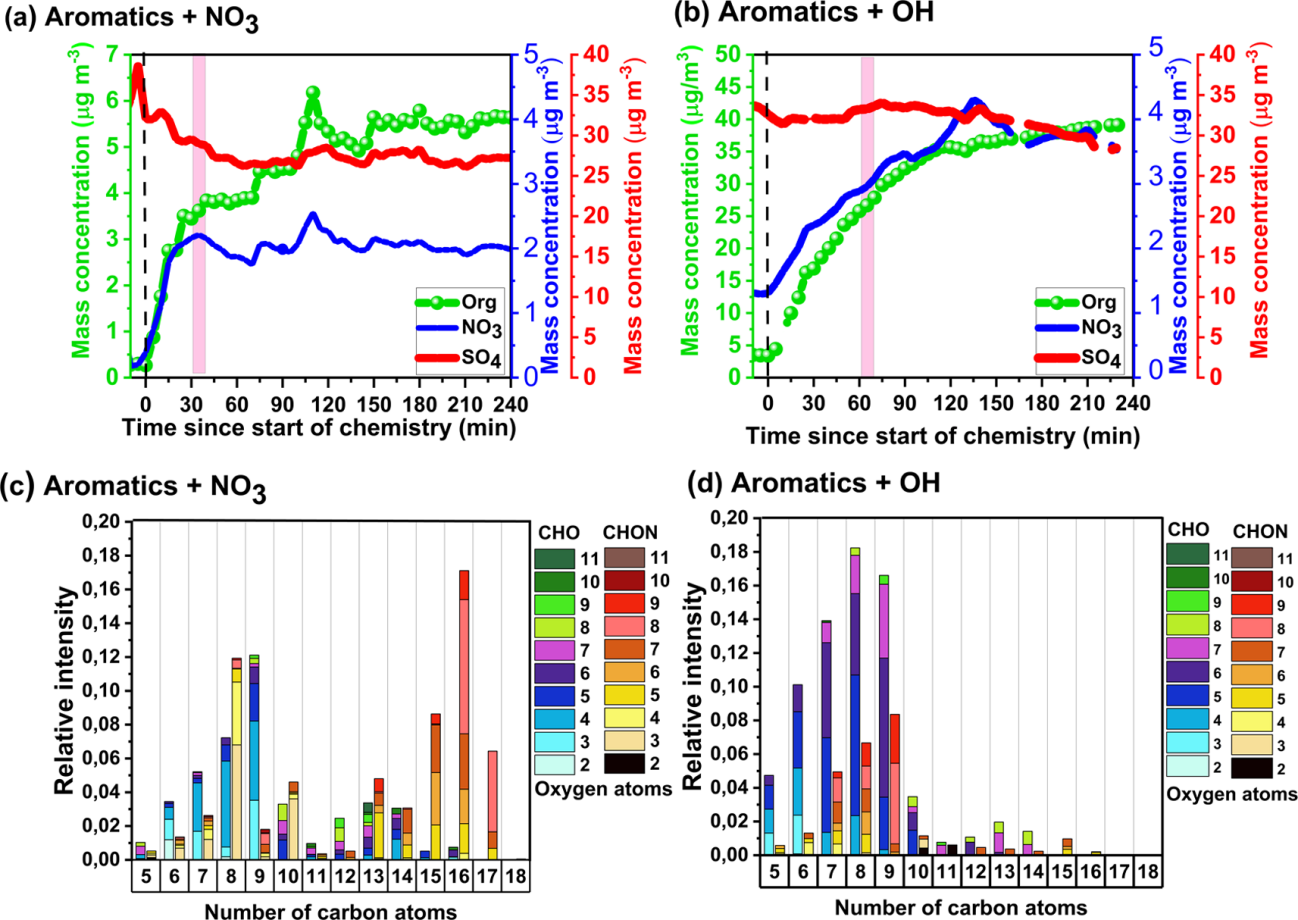
SOA mass evolution after the start of chemistry for (a) aromatics + NO_3_ and (b) aromatics + OH. Also included are the particle-phase nitrate and sulfate concentrations measured by the AMS. Note, the background of NO_3_^–^ is ∼1 μg m^–3^ in our OH experiments, which may come from incorporation of HNO_3_ left over from the NO_3_ experiments. The zero on the *x*-axis represents the time of the start of the reaction, i.e., when VOCs are first exposed to radicals. Pink shading denotes the time periods used to determine representative mass spectra for the aromatics + NO_3_ (c) and aromatics + OH (d) systems. These mass spectra are represented as carbon number distributions, with bins divided into CHO (left bar, blue-purple-green shading) and CHON (right bar, yellow-red-brown shading), and stacked vertically by number of oxygen atoms.

Figure 1a,b shows organics, sulfate, and nitrate concentrations measured by the AMS for the NO_3_ and OH experiment, respectively. In both sets of experiments, formation of organic mass is observed after the start of chemistry. However, the rate of SOA formation differs between the experiments, with formation occurring almost exclusively during the first 30 min for the NO_3_ experiment (Figure 1a), contrasting with steady formation over the entire 4 h for the OH experiment (Figure 1b). It should be noted that the initial rate of VOC consumption and SOA formation relates to different protocols used for radical introduction where a presumably large burst of NO_3_ radicals was formed in the starting vs a continuous formation of OH radicals. The formation rates of SOA in Figure 1a,b are consistent with the differences in VOC consumption shown in Figure S3. Additionally, the difference in the magnitude of SOA formed from each experiment relates to the concentration and identity of the VOCs available to react in the chamber. Because the same mixture was used for both experiments but only the ArHC–OH subset has significant reactivity with NO_3_, the OA concentrations obtained in these experiments are lower than in the OH experiments. The SOA yield from the OH experiment was 5.2%, which is higher than that from the NO_3_ system by more than a factor of 2. Further, Mutzel et al. (2021)^33^ reported that the SOA yield from cresol + NO_3_ was only 1% in the experiments conducted at lower organic mass concentrations (a factor of ∼5 lower than in our experiments). In contrast, we observed an aggregate SOA yield of 2% for the NO_3_ experiment. This variation in yield could be attributed to different partitioning behaviors under different organic mass concentrations, as well as the impact of a mixture of VOCs (i.e., multiple VOCs with interacting products) on the overall SOA yield.

### 3.2 SOA Composition

#### 3.2.1 SOA Composition Measurements by EESI-TOF

To highlight the detailed changes in the composition, the characteristic EESI-TOF mass spectra are binned according to the number of carbon atoms and stacked according to the number of oxygen atoms in Figure 1c,d, for the NO_3_ and OH experiment, respectively. For each carbon number, the column on the left indicates the observed CHO molecules and the column on the right indicates the observed CHON molecules. The molecular SOA composition in the NO_3_ experiment (Figure 1c) is dominated by nitrogen-containing species, with C_*x*_H_*y*_O_*z*_N molecules constituting ∼65.9% of the measured signal and the remainder (∼34.1%) comprising C_*x*_H_*y*_O_*z*_ molecules. Dimers (with carbon numbers C_12_ to C_18_) make up 54.2% of the signal, with the majority being C_*x*_H_*y*_O_*z*_N dimers constituting 42.3% of the total signal. In contrast, the composition of SOA formed from OH radicals is dominated by C_*x*_H_*y*_O_*z*_ molecules, constituting ∼75% of the total signal with the remainder (∼25%) coming from C_*x*_H_*y*_O_*z*_N species. Note, the dimer distribution peaks at C_16_ molecules while the other two most important dimers formed in the NO_3_ experiments (C_15_, C_17_) must come from reactions between pairs of different VOCs (e.g., C_8_ + C_7_ or C_8_ + C_9_). Table S3 shows the ratio of H-abstraction vs NO_3_ addition varies greatly with the molecular structure of the species. For less substituted alcohols, the H-abstraction is favored over NO_3_ addition but for highly substituted species such as trimethylbenzene, the NO_3_ addition is the dominant pathway. The H-abstraction pathway leads to an alkoxy radical, while the NO_3_-addition pathway leads to an RO_2_ radical. Therefore, we expect that the larger aromatic alcohols will form a larger fraction of dimers from RO_2_ radicals, which is consistent with our observations where there is higher prevalence of N-containing dimers with *C* > 15 in NO_3_ experiments. This highlights how the mixture of gases present will influence the amount of SOA formed, since they are dependent on the other oxidation products present. Similar results have been shown for mixtures of monoterpenes recently.^51,52^ Further, for the OH experiment, dimers (C_12_–C_18_) only make up a small fraction of the total measured contribution (∼6.5% in total for C_*x*_H_*y*_O_*z*_ (4.6%) and C_*x*_H_*y*_O_*z*_N (1.9%)), while the C_*x*_H_*y*_O_*z*_ and C_*x*_H_*y*_O_*z*_N monomers (C_5_–C_9_) comprise ∼64.7 and ∼22.5% of the total EESI mass flux, respectively. It should be noted that the method used to produce OH radicals (HONO photolysis) results in a steady presence of NO_*x*_ in the chamber. We observed the presence of approximately 1 ppbv of NO, with NO_2_ levels gradually increasing over time. This leads to a substantial fraction of CHON type species being formed in the particle phase, as observed. The remainder of the composition (∼6%) is composed of C_10_ (5.5%) and C_11_ (0.5%) species. Here, the importance of the mixed reaction products is smaller, given the smaller fraction of dimers, but odd carbon products (C_13_ and C_15_) again indicate the influence of mixed VOC reaction products.

Formation of SOA is governed by gas-particle partitioning of molecules possessing sufficiently low saturation vapor concentration to partition into the particle phase. The formation of low-volatility molecules occurs through oxidation reactions in the gas-phase and their subsequent radical reaction pathways. In reactions of ArHC–OH molecules with NO_3_ radicals, the dominant fate of RO_2_ radicals is either RO_2_ + RO_2_ or RO_2_ + NO_3_ reactions, because HO_2_ is not expected to be formed during these experiments. The radical balance in the chamber was modeled using F0AM to simulate the reactivity of first-generation RO_2_ radicals formed by the reaction of cresol with NO_3_ radicals (Figure S4a), where RO_2_ radicals are expected to react almost exclusively with other RO_2_ radicals forming self- or cross-reaction products (dimers). Recent work demonstrates the importance of RO_2_ + RO_2_ reactions as a pathway for dimer formation,^53−56^ which is likely the dominant source of dimers observed in Figure 1c. Dimers formed via this pathway will possess sufficiently low saturation vapor concentrations for condensation, resulting in rapid formation of SOA mass, consistent with the large fraction of dimers present in Figure 1c. This pathway is also likely responsible for the formation of mixed oxidation products, and their inclusion in the SOA.

In contrast, the OH experiments have significant concentrations of HO_2_ radicals and relatively lower concentrations of RO_2_ radicals because of a continuous injection of HONO instead of a single large burst of radicals as occurs in the NO_3_ experiments. This leads to continuous reactions of precursor VOCs + OH, rather than prompt consumption of all reactive VOCs. In addition, in the case of the OH experiments, we have NO and HO_2_ radicals available which will drive the RO_2_ chemistry toward RO_2_ + NO and RO_2_ + HO_2_ reactions. Figure S4b shows the simulated reactivity of a representative first-generation RO_2_ radical formed from toluene + OH, where the expected fate of RO_2_ radicals is dominated by reactions with HO_2_ and NO, with negligible RO_2_ + RO_2_ reactions. Therefore, the contribution of dimers formed in the gas phase will also be minor, consistent with the small dimer fraction observed in Figure 1d. The difference in modeled radical regimes present in each experiment (NO_3_ vs OH) provides an initial explanation of the monomer and dimer distributions present in Figure 1c,d, and also why the magnitude of cross VOC products is smaller for the OH chemistry system.

In addition to the differences in the detailed chemical composition observed in both experiments, these experiments differ also in terms of bulk elemental ratios. As measured by the EESI-TOF, the average O/C ratios are ∼0.45 and ∼0.72 for the NO_3_ and OH experiments, respectively, while the N/C ratios are ∼0.055 and ∼0.030 for the NO_3_ and OH experiments, respectively. A point to note here is that the N/C ratios in the NO_3_ and OH experiments differ by less than a factor of 2, despite having substantially different contributions of nitrogen containing species (C_*x*_H_*y*_O_*z*_N) to the total EESI signal in the respective experiments (as mentioned above in this section). This can be explained by the fact that most of the nitrogen in the NO_3_ experiment is contained in the dimers which have lower N/C ratio than monomers, whereas in the OH experiment all the nitrogen are found in the monomer species. As a result, despite having substantially different contributions of nitrogen containing compounds, N/C is not substantially different in the NO_3_ and OH experiments. Nevertheless, the elemental ratios from the EESI-TOF show some differences in SOA composition between NO_3_ and OH experiments, but because the EESI-TOF has ion-dependent sensitivities,^42,57^ a quantitative overview can only be obtained by analyzing the elemental ratios observed by the AMS.

#### 3.2.2 AMS SOA Composition

**Figure 2:**
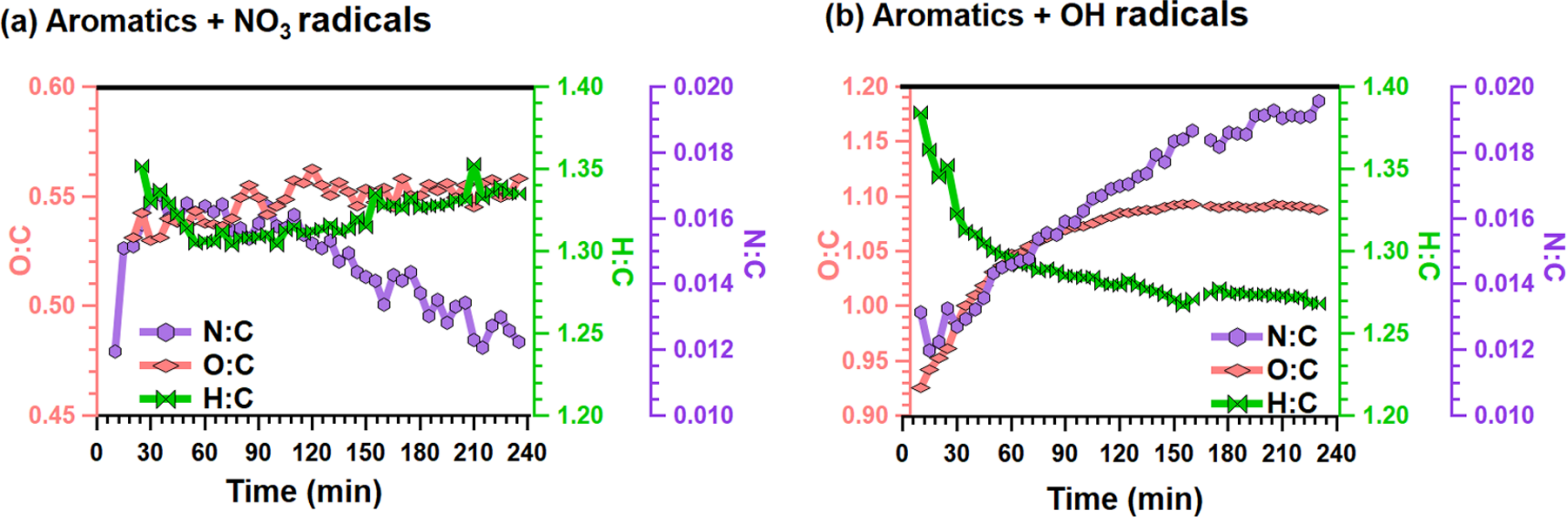
Time-series O/C, H/C, and N/C ratios obtained from the AMS for (a) NO_3_ experiment and (b) OH experiment.

Figure 2a,b shows the time series of elemental ratios (i.e., O/C, H/C and N/C) determined by the AMS^58,59^ for the NO_3_ and OH experiments, respectively. There is a considerable difference in the level of oxygenation for the NO_3_ (mean value of 0.54) vs OH (mean value of 1.01) experiments, which is consistent with the differences observed by the EESI-TOF between the OH and NO_3_ experiments. The N/C ratios measured by the AMS lie in the range (0.012–0.016) for the NO_3_ experiments and (0.012–0.019) for the OH experiment and are, in both the NO_3_ and OH experiments, a factor of 3–5 lower than those of the EESI (Figure S5). This is most likely the result of a combination of two factors: (1) breakdown of organonitrates to the inorganic ions NO^+^ and NO_2_^+^ in the AMS, which are then not included in the SOA organic mass calculation nor the bulk elemental ratios, and potentially (2) uncertainties in ion-specific response factors in the EESI-TOF.^60^ Nevertheless, the temporal experimental trends observed by the two instruments agree with each other for both the OH and NO_3_ experiments (Figure S5). As a comparison, the O/C ratios of logwood combustion emissions were reported to lie between 0.74 and 0.83 after 4 h of dark aging by NO_3_ radicals,^61^ whereas for single component systems, for example, SOA from m-xylene or toluene with OH radicals under high NO_*x*_ conditions were reported to lie between 0.68 and 0.72.^62^ In the experiments presented here, oxidation by the OH radicals systematically yields a higher level of oxygenation in SOA than does oxidation by the NO_3_ radicals. One explanation for the lower degree of oxygenation in the NO_3_ experiments is that the dominance of RO_2_ + RO_2_ results in the formation of dimers with comparatively lower O/C ratios while in the OH experiments, the first- and second-generation products can undergo further oxidation with OH leading to a higher degree of oxygenation. These explanations also explain the evolution of the O/C ratio with time, where there is a large increase in the O/C ratio in the OH experiment, and a slightly increasing value in the NO_3_ experiment within the first 2 h.

Beyond the evolution of the O/C ratio, the N/C ratio also continually evolves in both experiments. In the OH experiments, the N/C ratio gradually grows from 0.013 to 0.019 (SD = 0.002), which may be explained by the increased formation of organonitrates from the increasing NO_*x*_ availability in the chamber resulting from HONO photolysis. In the NO_3_ experiments, the N/C ratio measured by the AMS rapidly increases in the first 30 min and then steadily decreases from 0.016 to 0.012 (SD = 0.002) from 30 to 240 min. Similarly, the EESI-TOF N/C ratio shows a rapid increase and then a continuous decrease (Figure S5). This indicates that the chemical composition of the SOA in the NO_3_ experiments evolves with time even in the absence of large changes in OA mass concentration, and that these changes exert considerable influence on the bulk composition.

### 3.3 Evolution of the SOA Molecular Composition with Time

**Figure 3:**
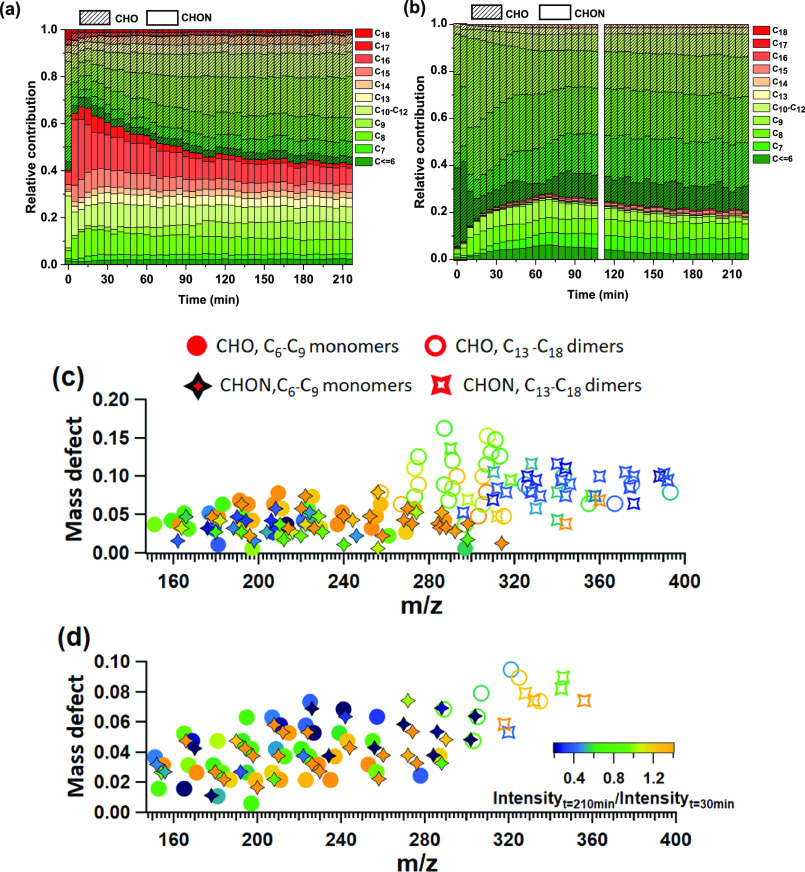
(a) Temporal evolution of fractional contributions from species in the aromatics + NO_3_ experiment color-coded by their carbon number (b) Same plot for the aromatics + OH experiment. (c) Mass-defect plot (exact mass minus nearest integer mass vs *m*/*z*) color-coded by ratio of intensity at 210 min to intensity at 30 min for the aromatics + NO_3_ system. Closed circles depict CHO C_6_–C_9_ monomers, whereas open circles depict CHO C_13_–C_18_ compounds. The closed and open diamonds depict CHON monomer and dimer species, respectively. (d) Mass-defect plot color-coded by ratio of intensity at 210 min to intensity at 30 min for the aromatics + OH system.

The ongoing changes in the bulk composition demonstrated in the previous section can be further interrogated by the EESI-TOF spectral evolution. Figure 3a,b shows the time-dependent evolution of the carbon number distributions (i.e., Figure 1c,d) for the NO_3_ and OH experiment, respectively, with C_*x*_H_*y*_O_*z*_ and C_*x*_H_*y*_O_*z*_N molecules distinguished by the shading pattern. For the NO_3_ experiment, the fractional contribution of dimer species (C_15_–C_18_) decreases with time (Figure S7), corresponding to increases in the fraction of both C_*x*_H_*y*_O_*z*_ (C_7_H_*y*_O_*z*_, C_8_H_*y*_O_*z*_, and C_9_H_*y*_O_*z*_) and C_*x*_H_*y*_O_*z*_N (C_9_) monomers. Figure S6 shows some key species that increase over the course of the experiment, including C_9_H_12_O_3_, C_9_H_14_O_4_, C_9_H_13_NO_7_, C_8_H_12_O_4,_ and C_7_H_8_O_3_. The total wall loss-corrected organic mass slightly increases between 30 and 240 min after the start of chemistry (Figure 1a) coinciding with the period during which the dimer decay is most prevalent, indicating there is likely minimal evaporation from the particle phase. Because the relative sensitivities of the EESI-TOF toward dimers vs monomers are not well constrained, the actual dimer and monomer fractions might be different from what is observed here. Considering the VOCs are completely consumed after ∼10 min during the NO_3_ experiments, the changes in particle-phase composition are likely driven by intra-particle reactions. The loss of dimers cannot be explained by evaporation because dimers are expected to have lower saturation vapor concentrations than their corresponding monomers and should not readily evaporate under our experimental conditions. Particle-phase reactions have also been shown to occur in α-pinene SOA derived from both NO_3_ and O_3_.^28,29,43^ In the experiments presented here, the main process governing the change in composition is the decay of dimers to form smaller molecules, specifically CHO and CHON monomers. This proposed dimer-to-monomer conversion differs from the particle-phase reactions observed in α-pinene + O_3_ experiments^28^ where the dominant process was a shift from higher carbon number species to lower carbon number species within monomers and dimers (and without substantial evidence of dimer-to-monomer conversion). The presence of NO_2_ and O_3_ in the chamber can lead to the formation of N_2_O_5_ which can hydrolyze quickly and can lead to the formation of NO_3_ ions that react with organic material in the particle phase to form nitrogen containing SOA species. However, the absence of a significant increase in particle phase NO_3_ suggests that the majority of changes observed in the SOA composition during the NO_3_ experiments are due to particle phase reactions, rather than later generation gas-phase chemistry involving N_2_O_5_. It is worth noting that the MCM in its current form does not include pathways for multi-generation NO_3_ chemistry.

Figure 3b shows the time evolution of the carbon number distribution for the OH experiment, showing much less change in the SOA composition than for the NO_3_ experiments (Figure 3a). Here, the fractional contribution from CHON species changes from ∼27% after ∼1 h to ∼19% after ∼4 h from the start of the experiment. Similarly, the fractional contribution of CHO species increases from ∼73 to ∼81% during the same time. The SOA composition from the OH oxidation (Figure 3b) is dominated by monomer CHO species with carbon number C_6_–C_9_. Although a slight increase in the fractional contributions from higher carbon number species (>C_9_) is observed with time, this is a minor contribution (increasing to a total of ∼4% at the end of experiment) in comparison with the NO_3_ experiments (∼55% at the beginning of the experiment and ∼27% at the end). However, this does not necessarily indicate the absence of particle-phase processes during OH oxidation, but rather that the overall SOA composition is dominated by the continuous formation of low-volatility material in the gas-phase which partitions into the particles, which could obscure the effects of particle-phase reactions in the OH system. Further, other studies have shown that multi-generation chemistry taking place in the gas-phase are responsible for the continued formation of SOA later in the experiment.^63^ This contrasts with the NO_3_ experiments, where all reactive VOCs are consumed within the first 10–15 min, and limited multi-generation chemistry is taking place.

Figure S8 shows the time series of selected CHON dimers (grouped by hydrogen number), which decay during the course of the NO_3_ experiment. There are a variety of different decay rates during the initial aging period, with some of the molecules (e.g., C_17_H_19_NO_*x*_) decaying much faster (decay rate = 0.034 min^–1^) than others (e.g., C_16_H_15_NO_*x*_; decay rate = 0.022 min^–1^). Additionally, the extent of the decay (i.e., fraction remaining) differs from molecule to molecule. To highlight these differences, Figure 3c,d shows mass-defect plots color-coded by the ratio of signal at 210 vs 30 min after the start of chemistry, that is, EESI (*t*_210_)/EESI (*t*_30_) for the NO_3_ (Figure 3c) and OH experiments (Figure 3d). Different symbols denote CHO and CHON monomers (C_6_–C_9_) and dimers (C_13_–C_16_).

For the NO_3_ experiment (Figure 3c), nearly all CHON dimers (open diamonds) exhibit EESI (*t*_210_)/EESI (*t*_30_) < 1, with most decreasing by more than 75%. This feature is not observed for CHO dimers, which range from a ∼30% increase to ∼30% decrease. CHON monomers are quite variable, ranging from a ∼75% decrease to a ∼75% increase. CHO monomers show a similar behavior, though relative to CHON they are more likely to show an increase (or smaller decrease). Figure 3a,c implies that in the NO_3_ experiments, CHON dimers decompose into smaller molecules, likely including both CHON and CHO. The observations cannot be explained by evaporation, which would result in lower carbon number dimers decaying faster than higher carbon number dimers. The opposite trend is observed here (higher mass compounds decay more quickly) and is therefore attributed to particle-phase decomposition reactions.

Overall, the SOA composition changes dramatically over the course of the NO_3_ experiment, where the initial composition is ∼25% monomers and ∼75% dimers, ultimately changing to ∼65% monomers and ∼35% dimers. Although variations in molecule-dependent EESI-TOF sensitivities make these compositional changes difficult to interpret quantitatively, they correspond well with the changes in the bulk N/C ratio from the AMS shown in Figure 2a. There, the N/C ratio decreases by ∼25% from *t* = 30 min to *t* = 210 min, and likely reflects the conversion of CHON dimers to CHO monomers, which in turn suggests that the changes observed in the EESI-TOF indeed reflect a considerable change in bulk composition. Although the AMS measurements do not provide a comparable means of assessing CHON dimers vs CHON monomers, given the similar behavior of CHO and CHON monomers, we consider it likely that the CHON dimer-to-monomer conversion likewise has a considerable impact on the bulk composition. To further probe this effect, the fraction of organonitrates to total OA was calculated from the AMS NO/NO_2_ ratio following the method described in Kiendler-Scharr et al. (2016).^64^Figure S9 indicates rapid organonitrate formation consistent with the observations by the EESI-TOF. Over the course of the experiment, however, the total organonitrate fraction remains stable in the AMS data. This contrasts with the EESI-TOF signal where the contribution of CHON compounds to total signal decreases from a maximum of ∼60 to ∼45% over the course of 3 h. This may reflect a somewhat higher sensitivity of the EESI-TOF toward dimers, where the largest changes in CHON/CHO are observed.

Figure 3d shows the mass defect plot color-coded by the ratio of the intensity at 210 min after the start of chemistry to the intensity at 30 min, that is, EESI (*t*_210_)/EESI (*t*_30_) for the OH experiment. Most of the molecules that are increasing during the experiment are typically more highly oxygenated species consistent with an increasing O/C ratio observed by both AMS and EESI-TOF. Additionally, there are increases in smaller molecules (C_6_–C_7_), which could come from multi-generational chemistry from smaller early generation molecules (e.g., first-generation oxidation products of benzene/toluene or fragmentation products of C_8_/C_9_ species). The majority of species that decrease with experimental time are C_9_H_12–14_O_4–7_ and C_9_H_11–15_O_5–8_N. These molecules could either react (e.g., fragment) in the particle phase or undergo repartitioning into the gas-phase as they react away in the gas-phase and reestablish equilibrium. Distinguishing between these processes is not possible in the current experiments because they occur simultaneously. Nonetheless, these results highlight, for both radical systems, the continuously evolving composition of SOA.

### 3.4 Decay Rates of Dimer Species in the NO_3_ Experiment

For the NO_3_ experiment, decay rates were calculated for C_*x*_H_*y*_O_*z*_N species that decayed to less than 50% of their maximum signal (e.g., C_7_ and C_8_ monomers and C_14_–C_17_ dimers) by fitting an exponential (*y* = *y*_0_ + *Ae*^*–kt*^) to the individual time series from the time of the maximum signal to the end of the experiment. The offset (*y*_0_) is included because the species examined here do not decay to zero, presumably due to the presence of isomers with different functionalities as also concluded by Pospisilova et al. (2020).^28^ The decay rate (*k*) determined by the fit is reported in min^–1^.

**Figure 4:**
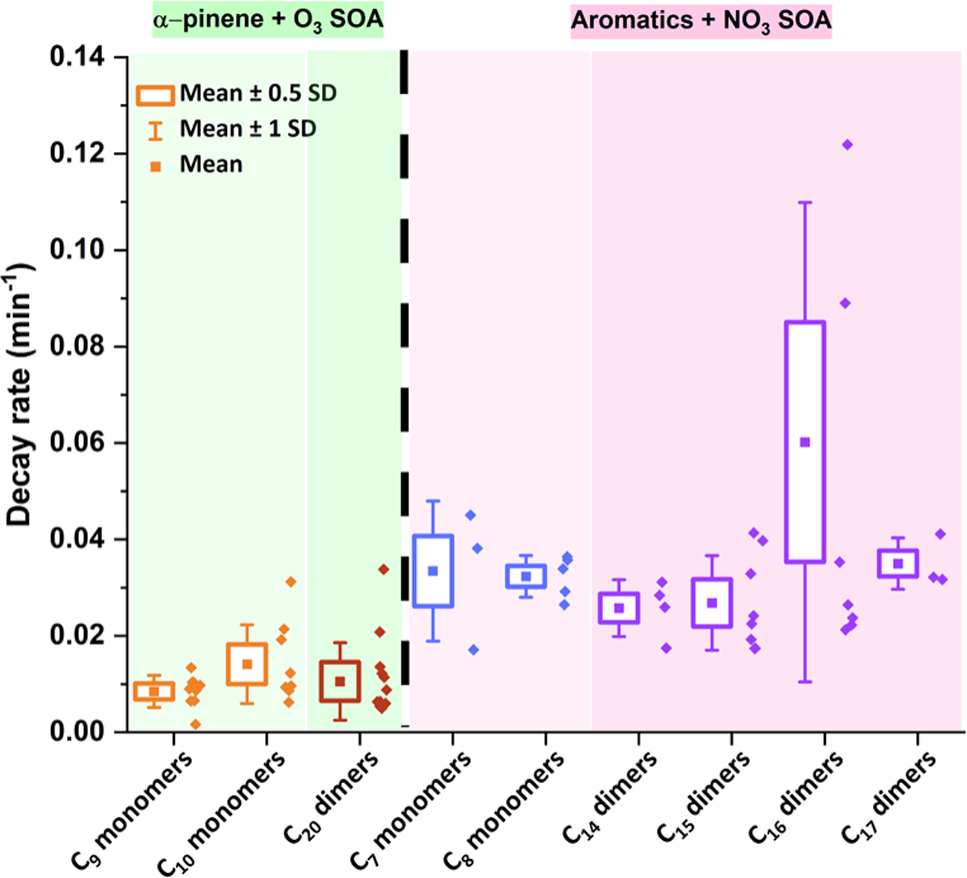
Box–whisker plots of decay rates calculated for C_*x*_H_*y*_O_*z*_ type C_9_–C_10_ monomers and C_20_ dimer species in α-pinene + O_3_ SOA^28^ highlighted by green areas. The points inside the respective box depict the mean rate of decay, whereas the diamonds adjacent to the boxed depict the spread of data. Similarly, the decay rates highlighted by pink areas are from C_*x*_H_*y*_O_*z*_N type C_7_–C_8_ monomers and C_14_–C_17_ dimers observed in the aromatics + NO_3_ system in this study.

The decay rates shown in Figure 4 are aggregated according to the number of carbon atoms present in the molecular formulae. The fastest decaying dimers observed were C_16_ species, which had an average decay rate of 0.060 min^–1^ (*n* = 8), of which the fastest decaying species were C_16_H_17_NO_4_, C_16_H_17_NO_5_, and C_16_H_19_NO_5_ (decay rates of 0.08, 0.12, and 0.14 min^–1^, respectively). By comparison, the average decay rates of C_17_, C_15,_ and C_14_ species were 0.034, 0.025, and 0.024 min^–1^, respectively. Figure 4 also shows the decay rates presented in Pospisilova et al. (2020)^28^ from α-pinene + O_3_ SOA. For both monomers and dimers, the decay rates in the aromatic + NO_3_ system are faster than those in the α-pinene + O_3_ system. This may be due to the slower SOA formation in the α-pinene + O_3_ system compared to the aromatic + NO_3_ system studied here. The decay rates reported in both studies should be interpreted as lower limits, due to the potential for production during the decay period; the potential bias increases as SOA formation extends later into the experiment. For instance, in the α-pinene + O_3_ experiment, the maximum SOA mass was reached after 90–120 min, while for the mixture used in this study, the maximum SOA mass is reached after 15–20 min. A second difference is that all monomer and dimer species included are C_*x*_H_*y*_O_*z*_ type compounds in the α-pinene + O_3_ system and C_*x*_H_*y*_O_*z*_N type compounds in the aromatic + NO_3_ system (C_*x*_H_*y*_O_*z*_ type compounds excluded here). This suggests that the presence of a nitrate functional group may render a molecule more prone to particle-phase decomposition. The fast decomposition/decay of dimers implies the dimer linkage is likely prone to decomposition. This would fit the scenario that dimers are largely formed from RO_2_ + RO_2_ chemistry, where the peroxy linkage may be unstable, favoring decomposition to the constituent monomers.

## 4 Implications

In the nocturnal atmosphere, the RO_2_ + RO_2_ reactions could be an important sink for RO_2_ radicals depending upon the availability of HO_2_ and NO_3_ radicals,^65^ and NO. Our study demonstrates, in the NO_3_ experiments, the importance of nitrogen containing dimer species presumably formed through RO_2_ + RO_2_ reactions leading both to mixed VOC oxidation products and fast formation of SOA. After formation, the NO_3_-derived SOA evolves rapidly, in particular through the decomposition of CHON dimers to CHO and CHON monomer species. In the OH experiments, the chemical composition evolves steadily throughout the experiment; however, given the experimental conditions, this is presumably due to continuous formation of organic mass through the gas-phase chemistry.

The observation of rapid changes in composition in the absence of further oxidant exposure as observed in the NO_3_ experiments means that care is needed in relating SOA composition measured under controlled laboratory conditions to field observations. Specifically, aligning the chemical age of the laboratory and ambient SOA is critical to draw accurate conclusions regarding the contribution of reactive species in the particle phase (e.g., the CHON dimers observed here in the NO_3_ experiments), which likely contain health-relevant peroxy functionalities. Further, this suggests that semi-continuous measurement systems may systematically underestimate the importance of such decay-prone molecules due to ongoing decomposition reactions during the collection stage.

Our results also show that the evolution of SOA from aromatic + NO_3_ is significantly faster than of biogenic + O_3_ SOA,^28^ implying that the identity of the precursor VOC and the oxidant affects the rate and extent of SOA evolution. This should, however, be determined by conducting comparison studies of SOA evolution formed from different precursor VOCs but under similar conditions and a range of different oxidants. In the ambient environment, the oxidation of VOCs will proceed at a much slower rate because of lower oxidant (OH and NO_3_) concentrations as compared to these chamber experiments, and the presence of both HO_2_ and NO will change the fate of RO_2_ radicals in the atmosphere. This will result in lower dimer concentrations due to the quadratic dependence of the dimer formation rate on the monomer RO_2_ radical concentration. Additionally, such fast decays would be difficult to observe because there would be a continued formation of all oxidation products due to a continuous exposure to radicals, as opposed to the NO_3_ experiments here where a single burst of NO_3_ radicals was used. This would create a near-constant source of the rapidly decaying molecules, and presumably they would decay after they partition to the particle phase. The fast decay reactions, hence, would not be easily observed in the atmosphere despite their effects on particle composition, underscoring the need for targeted laboratory studies, such as those performed here, to elucidate these reactions under controlled conditions.

## Supporting Information Available

Detailed description of experimental protocols; data analysis and interpretation; and wall loss correction methods (PDF)
