# Supplementary material for: Time-Resolved Molecular Characterization of Secondary Organic Aerosol Formed from OH and NO3 Radical Initiated Oxidation of a Mixture of Aromatic Precursors
Source: Environ Sci Technol. 2023 Jul 27;57(31):11572–82. doi: 10.1021/acs.est.3c00225 (PMC10413940; doi:10.1021/acs.est.3c00225)
Supplement: Supplementary file 1 — es3c00225_si_001.pdf [file es3c00225_si_001.pdf]

Supplement information

Time-resolved molecular characterization of secondary organic aerosol formed from the OH and NO_3 radical initiated oxidation of a mixture of aromatic precursors

Varun Kumar[α], Jay G. Slowik[*], Urs Baltensperger, Andre S.H. Prevot[*], David M. Bell[*]

Laboratory of Atmospheric Chemistry, Paul Scherrer Institute (PSI), 5232 Villigen, Switzerland

* Correspondence to: David M. Bell (david.bell@psi.ch), Jay G. Slowik (jay.slowik@psi.ch), Andre S.H. Prevot (andre.prevot@psi.ch)

[α] Now at Institut National de l'Environnement Industriel et des Risques, Parc Technologique ALATA, 60550, Verneuil en Halatte, France

The supplement information includes:

Text S1: Experimental Protocols
Text S2: AMS and EESI-TOF Data analysis
Text S3: Wall loss correction
Text S4: Calculation of OH exposure
10 figures
3 tables

Text S1: Experimental Protocols

In the NO_3 experiments, after the chamber was cleaned with dry air, the RH was increased to the desired level (~50% or ~90%). Then the ArHC + ArHC-OH mixture was injected into the chamber volumetrically (10 µl) and its concentration was monitored with the PTR-MS. Once the concentrations stabilized, O_3 was injected into the chamber (300 ppbv), followed by polydisperse ammonium sulfate ($(NH_4)_2SO_4$) seed aerosol in the size range of 16-650 nm with a number size distribution mode at 60-80 nm, to yield a concentration of ~ 50-60 µg m^{-3} as measured by the SMPS. After stabilization of all precursors and seed particles (~10-30 min), a burst of NO_2 (1,000 ppmv, Messer, 99% purity) was injected into the chamber, reaching concentrations of ~500 ppbv. The burst of NO_2 leads to the generation of NO_3 radicals and N_2O_5. After the injection of NO_2 was complete, the chamber was stirred by injecting zero air into the chamber (100 L min^{-1} for ~1 min). The experiments lasted 4 hours after the injection of NO_2.

For the OH experiments, the VOC mixture and seed aerosol were injected using the same protocols as described above. HONO was used as an OH source, which was generated by mixing constant flows of 10 mM H_2SO_4 (95–97 %, Merck) and 2 mM $NaNO_2$ (≥ 99.0 %, Fluka) solutions, regulated by a programmable peristaltic pump (REGLO Digital MS-4/8, IDEX Health & Science GmbH, Germany). These liquids reacted in a custom-made vessel to produce HONO vapors which were carried to the chamber by passing zero air (0.5 L min^{-1}) through the vessel [1,2]. HONO was continuously injected into the chamber, and and OH radicals were produced by irradiating the chamber with a set of 80 UV lights (100 W, Cleo Performance, Philips) with emission intensity peaking at ~368 nm [2]. Photochemistry was initiated by turning on these lights, and the experiments lasted 4 hours thereafter. Blank experiments were conducted for both NO_3 and OH systems and no substantial formation of organic mass was observed. Table S2 summarizes the experimental conditions of all experiments conducted.

Text S2: AMS and EESI-TOF Data analysis

The integer mass resolution (UMR) data acquired from the AMS were analyzed using the SQUIRREL data analysis toolkit (version 1.59) programmed in IGOR Pro 6.37 software (Wavemetrics, Inc., Portland, OR, USA). High-resolution data were analyzed using the PIKA (version 1.19) algorithm following peak fitting [3] for mass to charge ratios *m/z* 12 to *m/z* 120**.**

The EESI data analysis was performed using Tofware version 2.5.7 (Tofwerk AG, Thun, Switzerland). The original data were acquired at 1 Hz resolution and were later averaged to 10 seconds on which the high-resolution peak fitting was performed for *m/z* 145-400 and the fitted peaks were assigned molecular formulae with carbon numbers ranging from C_5-C_{18}. It has been shown that small molecules (C_{2-4}) are present in SOA [1,2], however, no such peaks were identified here, which may result from the limited mass transmission in the EESI-TOF below 145 *m/z.* Regular background measurements were conducted every 5 minutes (i.e., 4 minutes of chamber air and then 1 minute of background), by sampling aerosols through a particle filter. Reported signals are calculated as the difference of unfiltered chamber air minus the average of background measurements immediately before and after each chamber measurement period. After background subtraction, the data were averaged to 5 minutes for further analysis and presentation of results. The measured EESI signal (ions s^{-1}) was converted to a

mass flux to the detector in attograms per second (ag s^{-1}) by using the following formula, applied on an ion-by-ion basis:

$$EESI(ag\ s^{-1}) = \frac{EESI(Hz) \times MW \times 10^{18}}{6.023 \times 10^{23}} \qquad (S1)$$

Note that this detector-based mass flux rate differs from the mass flux rate into the instrument due to uncertainties in molecule-dependent extraction and ionization efficiencies[4].

Text S3: Wall-loss correction

A size dependent wall-loss correction was applied to organics, nitrate and sulfate. The mass size distribution of sulfate was measured by the AMS throughout the experiment and was converted to effective number concentrations assuming a sulfate effective density of 1.79 g cm^{-3}. A size-dependent particle–wall deposition coefficient was then obtained by fitting a first-order exponential decay to the measured particle number concentration decay in logarithmically spaced bins ranging from 150-950 nm. The particle wall-loss correction factors applied on organics, nitrate and sulfate were obtained from two size bins. The first size bin selected was from 250-450 nm, representing the mode of the organic and nitrate size distribution, and the second bin was selected from 650-850 nm, representing the mode of the sulfate size distribution. The particle wall-loss is defined by the following formula:

$$\frac{dN}{dT} = -K_{coag}\, N^2 - K_{wall} N \qquad (S2)$$

In Equation S2, N is the particle number concentration and K_{coag} is the coagulation coefficient (5 × 10^{-10} s^{-1}). From this equation, the number of particles lost to the walls relative to coagulation is determined as a function of time. The mass of the particles lost to the wall is used to determine the wall loss correction factor $M_{correction}$ = ($M_{measured}$ + $M_{wall\ loss}$) / $M_{measured}$. The wall loss correction factors obtained for organics (250-450 nm) and sulfate (650-850 nm) are shown in Fig. S10.

Text S4: Calculation of OH exposure

The integrated OH radical concentrations or OH exposure was calculated based on different reactivities of two VOCs. In our case we used 9-fold deuterated butanol (d9-butanol) and toluene [5]. The OH rate constants of these compounds were taken as $k_{OH,but}$ =3.14 × 10^{-12} cm^3 $molec^{-1}$ s^{-1} and $k_{OH,tol}$ = 5.48 × 10^{-12} cm^3 $molec^{-1}$ s^{-1}[6], respectively. The integrated OH exposure is expressed as

$$OH\ exposure\ = \frac{\ln\left(\frac{d9-butanol}{toluene}\right)_{t=0} - \ln\left(\frac{d9-butanol}{toluene}\right)_{t=240min}}{k_{OH,but} - k_{OH,tol}} \qquad (S3)$$

The OH exposure was approx. 5.67×10^7 molec cm^{-3} h which is equivalent to 56.7 hours of ageing in the ambient atmosphere assuming a global average OH radical concentration of 1×10^6 molec cm^{-3}.

Figures

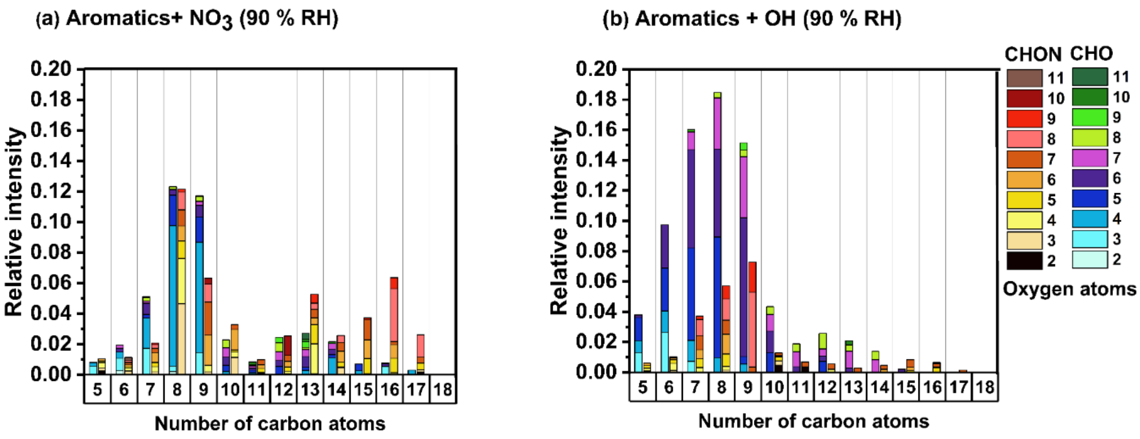

Figure S1: Carbon number distributions, with bins divided into CHO (left bar, blue-purple-green shading) and CHON (right bar, yellow-red-brown shading), and stacked vertically by the number of oxygen atoms for **(a)** aromatics+NO_3 system (90% RH), and **(b)** aromatics + OH system (90% RH).

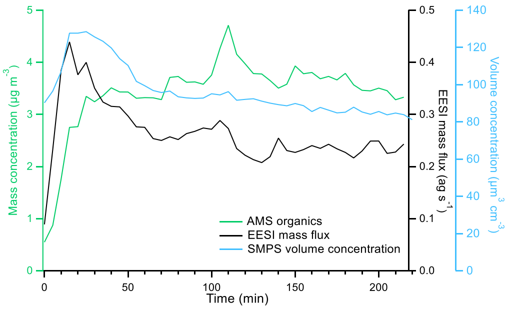

Figure S2: Comparison of the total EESI mass flux (ag s^{-1}) with total organic mass (in µg m^{-3}) measured by the HR-AMS and SMPS volume concentration in the NO_3 experiment. The time-series are not wall-loss corrected. The SMPS volume includes organics as well as inorganics such as sulfate and nitrate and water as well.

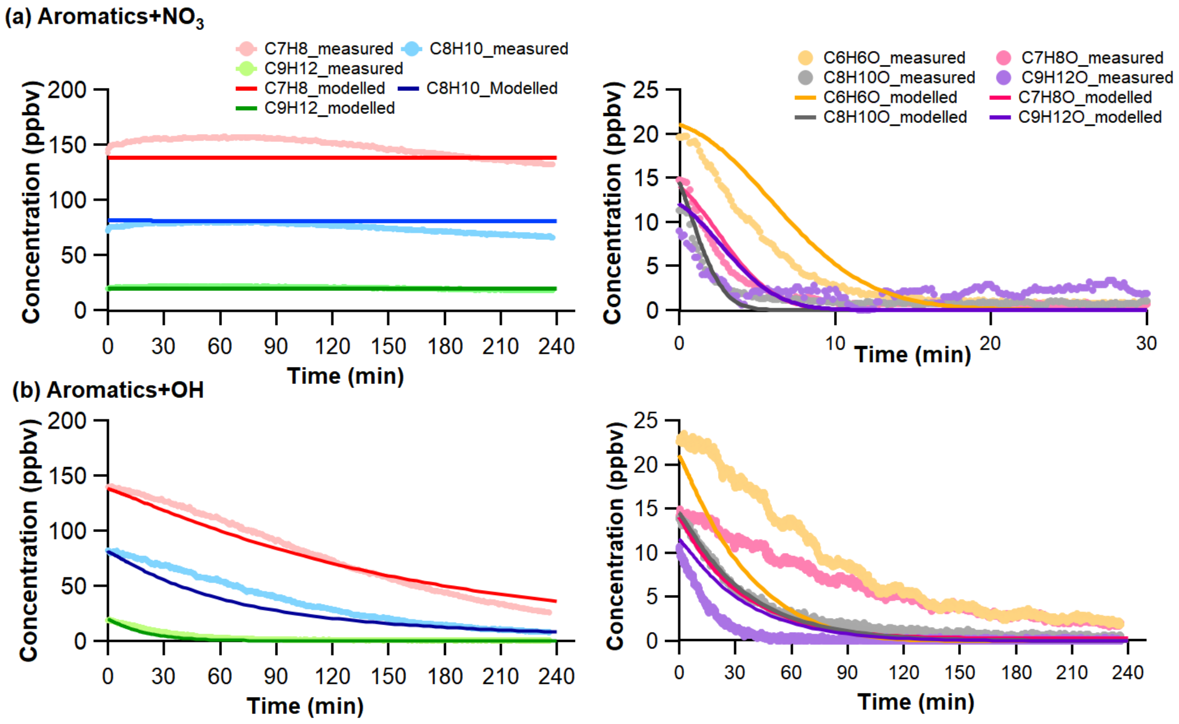

Figure S3: (a) Modelled and measured time series of various precursor VOCs used in the mixture for the NO_3 radical experiment. It is clear from the figure that ArHC compounds (left upper panel) essentially remain unreacted whereas ArHC-OH compounds (right upper panel) react quickly and almost get exhausted within the first 15-20 minutes after exposure to radicals. Zero on the x-axis corresponds to time when VOCs are first exposed to radicals. **(b)** Modelled and measured time series of various precursor VOCs used in the mixture for the OH radical experiment. In contrast to the NO_3 experiments, here both ArHC and ArHC-OH compounds react with OH radicals.

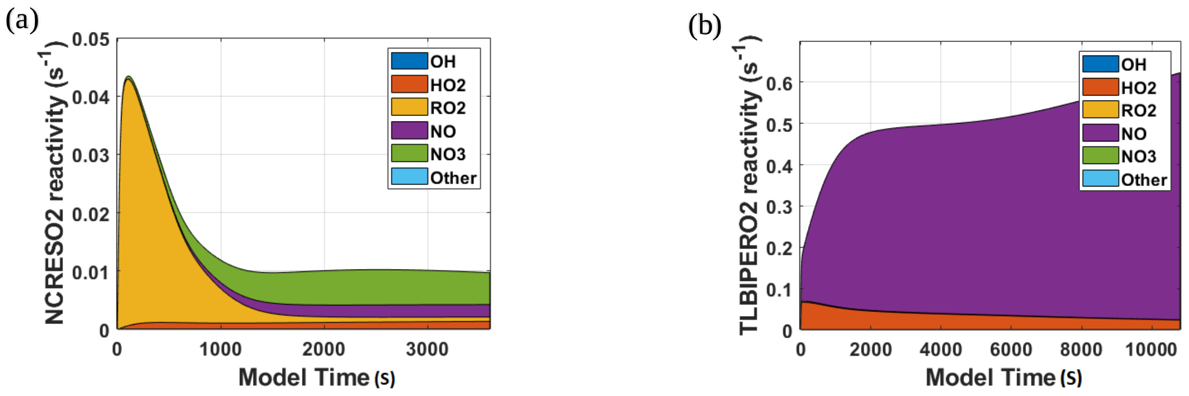

Figure S4: (a) F0AM Modelled reactivity of a representative first-generation cresol RO_2 radical formed by NO_3 reaction with cresol. The x-axis depicts the model run time in seconds. The model is run until 3600 seconds. The simulated reactivity shows that these RO_2 radicals reacts almost exclusively with other RO_2 radicals (orange) in the first 1000 seconds which is the time frame within which almost all VOCs get consumed. **(b)** Modelled reactivity of a representative first-generation toluene RO_2 radical formed by OH reaction with toluene. This shows that these RO_2 radicals predominantly react with HO_2 and NO.

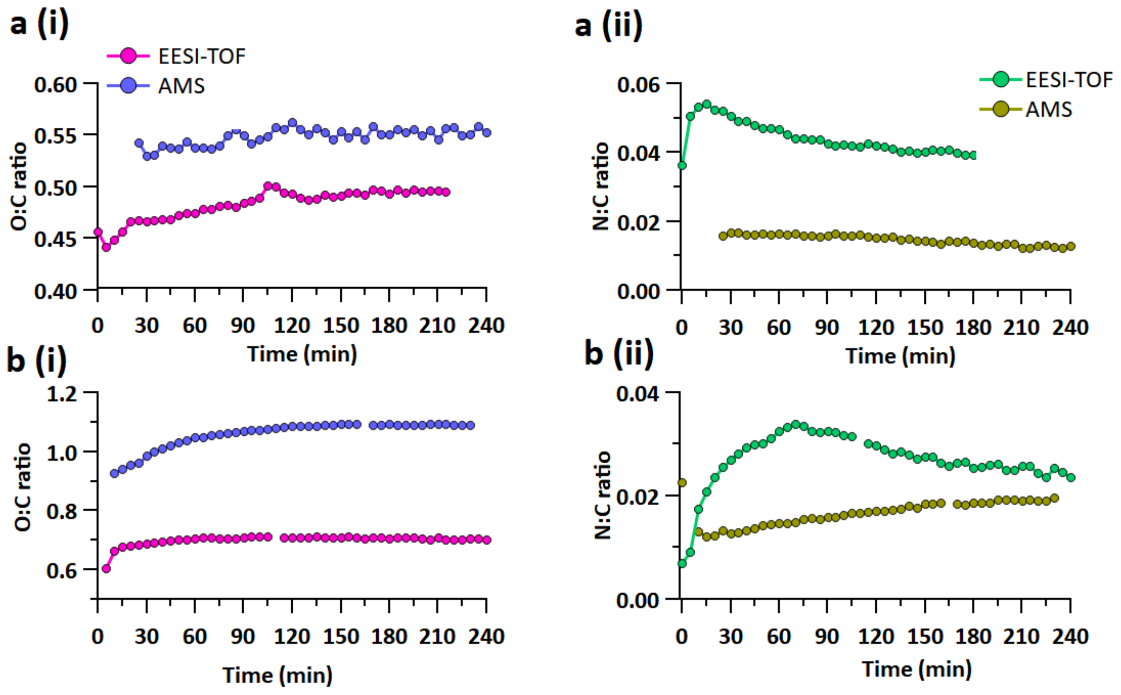

Figure S5: **(a)** O:C and N:C ratios measured by the EESI-TOF and the AMS during the NO_3 experiment. **(b)** O:C and N:C ratios measured by the EESI-TOF and the AMS during the OH experiment.

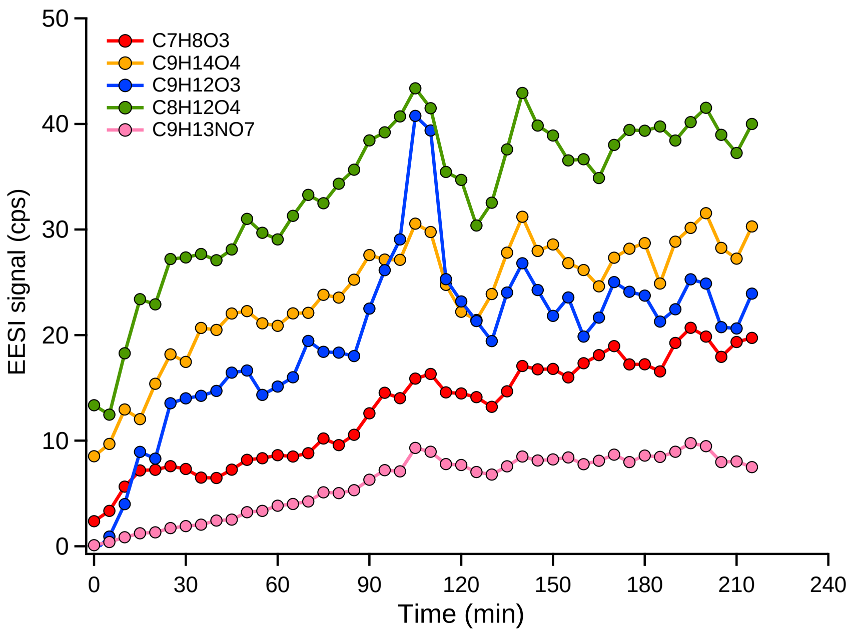

Figure S6: Time series of selected monomer species exhibiting an increase over time during the NO_3 experiments. The majority of increasing species are CHO species dominated by C_9 molecules, but some CHON species are observed as well.

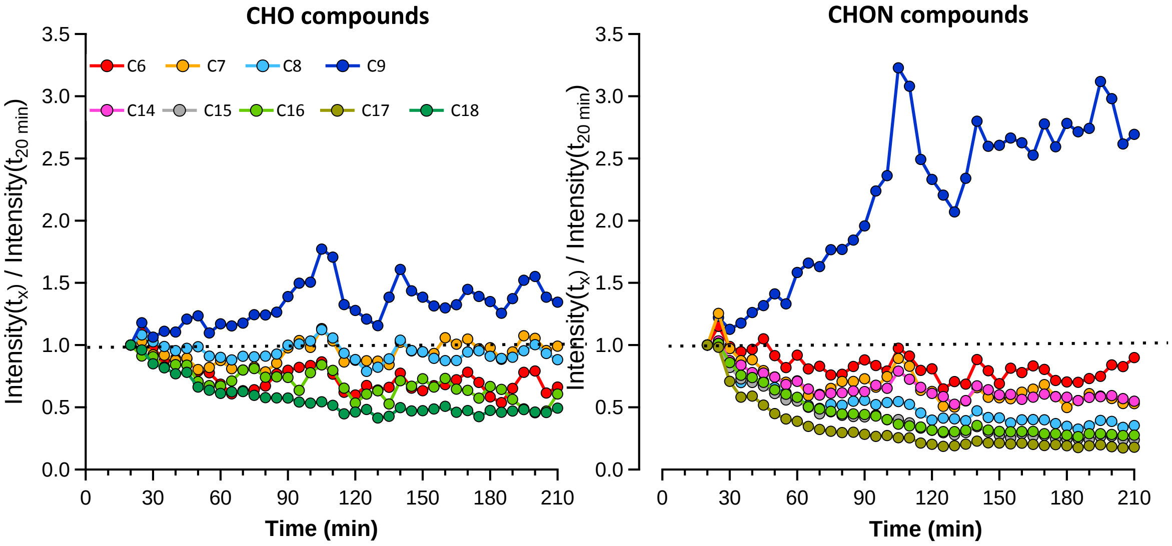

Figure S7: Intensity of C_6-C_9 monomers and C_{13}-C_{18} dimers measured as a ratio of intensity at *t*=20 min to time=*t* min (CHON compounds on the left and CHO compounds on the right) from the NO_3 experiment.

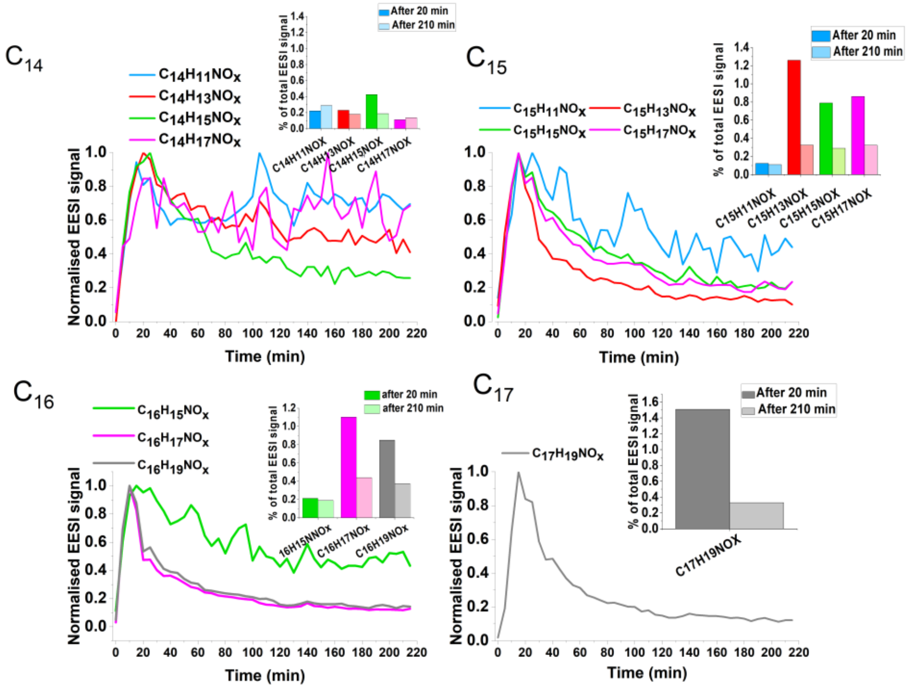

Figure S8: Intensity of C_{14}-C_{17} CHON dimers grouped by hydrogen numbers from the NO_3 experiment. The time series are normalized to their maximum values. The bars in the inset show the relative contributions of each group of dimers to the total EESI signal at 20 min and at 210 min thus showing their relative importance as a function of time.

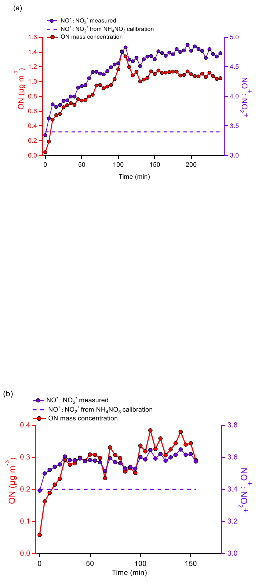

Figure S9: The calculated mass concentration of organo-nitrates (ON) (red) along with the NO^+:NO_2^+ measured (blue) as well as the one calculated during the NH_4NO_3 calibration (dashed violet line) during the NO_3 experiment **(a)** and OH experiment **(b)**.

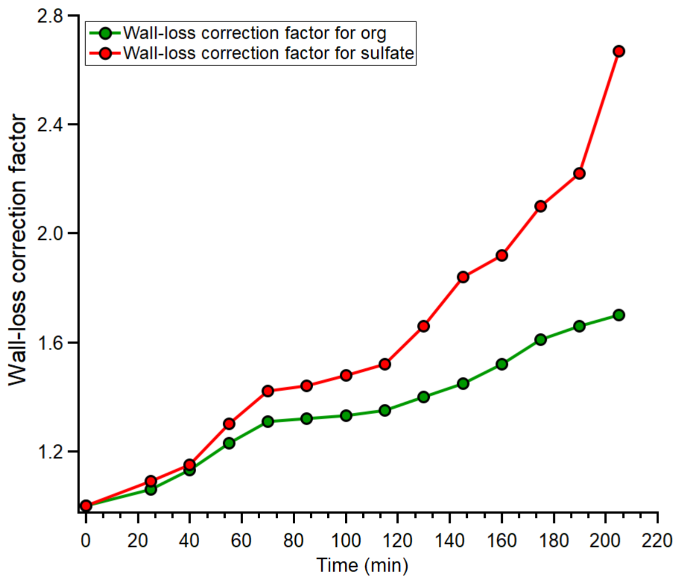

Figure S10: Wall-loss correction factors calculated for organics and sulfate.

Tables

Table S1: Approximate concentrations of the VOCs injected into the chamber in the experiments.

VOC	Concentration (ppbv)
Toluene (C_7H_8)	135
Ethylbenzene (C_8H_{10})	40
p-Xylene (C_8H_{10})	40
1,3,5 Trimethyl benzene (C_9H_{12})	20
Phenol (C_6H_6O)	18
Cresol (C_7H_8O)	12
2,6-Dimethyl phenol ($C_8H_{10}O$)	12
2,4,6-Trimethyl phenol ($C_9H_{12}O$)	12

Table S2: Experimental parameters for all experiments.

Experiment #	Oxidant	Maximum SOA (μg m^{-3})	RH (%)	Temp (K)	Mass spectrometers oparational	NO_2 (ppbv)	O_3 (ppbv)	VOC injected (ppbv)	HONO (ppbv)	Seed aerosol (μg m^{-3})
**1**	NO_3	3	50%	293	EESI-TOF, AMS & PTR-MS	~1000	~300	~300	None	50-60
**2**	NO_3	5	90%	294	EESI-TOF, AMS & PTR-MS	~1000	~300	~300	None	50-60
**3**	OH	24	50%	292	EESI-TOF (only 1hr) & AMS	None	None	~300	~50	50-60
**4**	OH	22	90%	295	EESI-TOF (only 2 hr) & AMS	None	None	~300	~50	50-60
**5**	OH	30	50%	293	EESI-TOF & PTR-MS	None	None	~300	~50	50-60

*volumetric addition

Table S3: Branching ratios of NO_3 addition vs. H-abstraction for ArHC-OH molecules used in our experiments.

Species name	Molecular formula	NO_3 addition	H-abstraction	MCM reference
phenol	C_6H_6O	0.26	0.74	MCM Website PHENOL (leeds.ac.uk)
cresol	C_7H_8O	0.61	0.39	MCM Website CRESOL (leeds.ac.uk)
2,3-dimethylphenol	$C_8H_{10}O$	0.61	0.39	MCM Website OXYLOL (leeds.ac.uk)
2,4,6-trimethymphenol	$C_9H_{12}O$	0.79	0.21	MCM Website TM135BZOL (leeds.ac.uk)

References

(1) Bruns, E. A.; Krapf, M.; Orasche, J.; Huang, Y.; Zimmermann, R.; Drinovec, L.; Močnik, G.; El-Haddad, I.; Slowik, J. G.; Dommen, J.; Baltensperger, U.; Prévôt, A. S. H. Characterization of Primary and Secondary Wood Combustion Products Generated under Different Burner Loads. *Atmos. Chem. Phys.* **2015**, *15* (5), 2825–2841. https://doi.org/10.5194/acp-15-2825-2015.

(2) Platt, S. M.; El Haddad, I.; Zardini, A. A.; Clairotte, M.; Astorga, C.; Wolf, R.; Slowik, J. G.; Temime-Roussel, B.; Marchand, N.; Ježek, I.; Drinovec, L.; Močnik, G.; Möhler, O.; Richter, R.; Barmet, P.; Bianchi, F.; Baltensperger, U.; Prévôt, A. S. H. Secondary Organic Aerosol Formation from Gasoline Vehicle Emissions in a New Mobile Environmental Reaction Chamber. *Atmos. Chem. Phys.* **2013**, *13* (18), 9141–9158. https://doi.org/10.5194/acp-13-9141-2013.

(3) DeCarlo, P. F.; Kimmel, J. R.; Trimborn, A.; Northway, M. J.; Jayne, J. T.; Aiken, A. C.; Gonin, M.; Fuhrer, K.; Horvath, T.; Docherty, K. S.; Worsnop, D. R.; Jimenez, J. L. Field-Deployable, High-Resolution, Time-of-Flight Aerosol Mass Spectrometer. *Anal. Chem.* **2006**, *78* (24), 8281–8289. https://doi.org/10.1021/ac061249n.

(4) Lopez-Hilfiker, F. D.; Pospisilova, V.; Huang, W.; Kalberer, M.; Mohr, C.; Stefenelli, G.; Thornton, J. A.; Baltensperger, U.; Prevot, A. S. H.; Slowik, J. G. An Extractive Electrospray Ionization Time-of-Flight Mass Spectrometer (EESI-TOF) for Online Measurement of Atmospheric Aerosol Particles. *Atmos. Meas. Tech.* **2019**, *12* (9), 4867–4886. https://doi.org/10.5194/amt-12-4867-2019.

(5) Barmet, P.; Dommen, J.; DeCarlo, P. F.; Tritscher, T.; Praplan, A. P.; Platt, S. M.; Prévôt, A. S. H.; Donahue, N. M.; Baltensperger, U. OH Clock Determination by Proton Transfer Reaction Mass Spectrometry at an Environmental Chamber. *Atmos. Meas. Tech.* **2012**, *5* (3), 647–656. https://doi.org/10.5194/amt-5-647-2012.

(6) Atkinson, R.; Arey, J. Atmospheric Degradation of Volatile Organic Compounds. *Chem. Rev.* **2003**, *103* (12), 4605–4638. https://doi.org/10.1021/cr0206420.
